# 

**Published:** 1820-06

**Authors:** 


### MONTHLY CATALOGUE OF MEDICAL BOOKS.

The First Lines of the Practice of Surgery; designed as an Introduction for Students, and a concise Book of Reference for Practitioners. By Samuel Cooper, late Surgeon to the Forces, &c. Vol. II. (which completes the Work). With several Plates, price 15s. boards.

A History of the Epidemic Fever which prevailed in Bristol, during the Years 1817, 1818, and 1819. Founded on Reports of St. Peter's Hospital and the Bristol Infirmary. By J. C. Prichard, M.D. 5s. boards.

Exposition of Elementary Principles especially concerned in the Preservation of Healthiness, and Production of Distempers, amongst Mariners, Travellers, and Adventurers, in Tropical, Variable, and Unkindly Climates. With Miscellaneous Illustrations of Prophylactical Administration. By Andrew Simpson, Surgeon, Glasgow. 18s. boards.

A Dictionary of Practical Surgery; comprehending all the most interesting Improvements up to the present Period. By Samuel Cooper, Esq. Third Edition, enlarged, price 1l. 4s.

### *TO CORRESPONDENTS.*

*Several original Communications, which we received too late to enable us to insert them in the present Number, will appear in the next. We regret, especially, this delay in the publication of that of* Dr. MACLEOD, *from the very interesting nature of the subject of it; but it is necessary that the first part of the Journal should pass the press very early in the month, after which the arrangement of the work will not admit of the insertion of original articles before the ensuing month.*

